# Impact of e-cigarette vaping aerosol exposure in pregnancy on mTOR signaling in rat fetal hippocampus

**DOI:** 10.3389/fnins.2023.1217127

**Published:** 2023-06-28

**Authors:** Jehoon Lee, Marcus R. Orzabal, Vishal D. Naik, Jayanth Ramadoss

**Affiliations:** ^1^Department of Veterinary Physiology and Pharmacology, Texas A&M University, College Station, TX, United States; ^2^Department of Obstetrics and Gynecology, C.S. Mott Center for Human Growth and Development, School of Medicine, Wayne State University, Detroit, MI, United States; ^3^Department of Physiology, School of Medicine, Wayne State University, Detroit, MI, United States

**Keywords:** pregnancy, development, fetal, nicotine, brain, vaping

## Abstract

Electronic cigarette (e-cig) use during pregnancy has become a major health concern in recent years and many view them as less harmful and may help quit or reduce combustible cigarettes. Implementing a state-of-the-art engineered vaping system, comprising an atomizer similar to those sold in vape shops, we aimed to utilize a translational e-cig inhalation delivery method to provide crucial information on the impact of prenatal e-cig aerosols on the developing brain hippocampal mTOR system in a rat model system. Gestational e-cig vaping significantly increased P-mTOR levels (*p* < 0.05) in the rat fetal hippocampi in the nicotine group (comprising of VG/PG + nicotine) compared to the control and the juice (comprising of VG/PG) groups. Total mTOR expression was not different among groups. Immunofluorescence imaging demonstrated P-mTOR was detected exclusively in the granule cells of the dentate gyrus of the fetal hippocampus. E-cig did not alter DEPTOR, but RAPTOR and RICTOR were higher (*p* < 0.05) in the Nicotine group. Gestational e-cig vaping with nicotine increased (*p* < 0.05) the activity and expression of 4EBP1, p70S6K, but decreased (*p* < 0.05) P-PKCα in the fetal hippocampi. In summary, dysregulation of mTORC1 and the related mTORC2, their activity, and downstream proteins together may play a critical role in e-cig-vaping-induced neurobiological phenotypes during development.

## Introduction

The rise of e-cig use in the past decade is a growing health concern, especially during pregnancy as many perceive e-cigs as a safer alternative to combustible cigarettes (Hajek et al., 2014). E-cigs are readily available in the market and have rapidly gained popularity. The United States Surgeon General warns that exposure to tobacco products, like e-cigarettes (e-cigs), during pregnancy may result in damaging and life-long consequences for the offspring (National Center for Chronic Disease Prevention and Health Promotion, 2014). National estimates of e-cig use among pregnant and nonpregnant women of reproductive age between 2014 and 2017 in the US was around 3.6% (Liu et al., 2019). Another study in a similar time frame estimates the use of e-cigs in pregnant population to have increased from 1.9% in 2016 to 3.18% in 2018 (Obisesan et al., 2020). Further, a recent study shows low fetal birth weight to be prevalent among those who use e-cigs in pregnancy compared to those who quit entirely (Regan and Pereira, 2021). Others have reported a paucity of research on vaping in pregnancy (Calder et al., 2021).

E-cig liquids primarily consist of a juice comprising of vegetable glycerin/propylene glycol base (VG/PG), flavorings, and nicotine (FDA, 2020) and typically include a handheld battery that rapidly heats a metal coil to aerosolize the e-cig liquid. Many aspects of e-liquids are not standardized by industry manufacturers, including the ratio of VG to PG, leading consumers to choose proportions based on their personal preferences (Smith et al., 2020). Although ingested juice is generally regarded as safe, aerosolized juice has not been classified as safe, especially in pregnancy (Qu et al., 2018; Berkelhamer et al., 2019; Frazier et al., 2021). Nicotine in vapor form has been reported to have a range of effects on reproductive vascular function, fetal and child development such as low birth weight, increased blood pressure and increase in pro-inflammatory serum markers, and nitrite oxide in the newborn (Suzuki et al., 1980; Aubert et al., 1987; Mohsenzadeh et al., 2014). Studies also report those who are already addicted to nicotine and who become pregnant may turn to e-cigs more than ever before (Obisesan et al., 2020).

Although the data on e-cig vaping and developing brain has been limited, the fetal brain has been demonstrated as a major target of prenatal e-cig exposure. E-cig exposure has been reported to disrupt mouse blood brain barrier and induce proinflammatory cytokines (Schmidt, 2020) in postnatal brain and induces long-term neurovascular changes and worsening behavioral outcomes (Sabrina Rahman Archie et al., 2023). E-cig exposure downregulates autophagy-related gene expression via DNA hypermethylation in neonatal brain in rat model (Walayat et al., 2021). Brain Mammalian Target of Rapamycin (mTOR) has emerged at the node of many fields of study. mTOR plays an integral role in modulating brain anabolic and catabolic activities, including protein synthesis and autophagy. mTOR is a 289-kDa serine/threonine protein kinase that makes up the catalytic subunit of two individual complexes, known as mTOR Complex 1 (mTORC1) and mTOR Complex 2 (mTORC2; Carabulea et al., 2023). mTORC1 plays a major role in multiple cellular signals to sense the availability of nutrients, growth factors, and energy to promote cellular growth and catabolic activity during stress, whereas less is known about mTORC2 (Carabulea et al., 2023). During fetal and neonatal brain development, mTOR signaling system plays critical roles in neuron differentiation, growth, and neuronal proliferation, and in homeostasis of stem cell and progenitor cell population (Easley et al., 2010; Lee, 2015), and its disruption has been associated with smaller brain size with fewer neurons and neural progenitors (Kim, 2015), translation dysregulation, and altered neuroprotection mediated by autophagy (Carabulea et al., 2023). Very little is known about effects of e-cig vaping on the mTOR system in the developing brain, and we herein aimed to discern how e-cig vaping may affect fetal hippocampal mTOR signaling in order to further elucidate developmental hippocampal effects. We hypothesized that gestational e-cig vaping exposure will dysregulate mTOR level/activity and disrupt the activity of proteins within its associated systems, mTORC1 and mTORC2, as well as its related downstream pathways in the fetal hippocampus.

## Materials and methods

### Animals

All experimental procedures were in accordance with National Institutes of Health guidelines (NIH Publication No. 85–23, revised 1996) and approved by the Institutional Animal Care and Use Committee at Texas A&M University. Timed-pregnant Sprague Dawley rats purchased from Charles River (Wilmington, MA) were housed in a temperature-controlled room (23°C), with a 12:12-h light–dark cycle. Animals were randomly assigned to pair-fed control (Control; *n* = 5 dams), pair-fed Juice without Nicotine (Juice; *n* = 5 dams), or Juice with Nicotine (Nicotine; *n* = 4 dams) groups on gestational day (GD) 5. All animals were weighed prior to the start of treatments. Control and Juice animals were yoked with Nicotine animals of similar weight for the duration of the study. Nicotine group animals received an *ad libitum* diet and daily feed intakes were measured. Control and Juice group animals were fed the same amount of diet consumed by Nicotine group animals. All animals had free access to water.

### E-cig vaping

The e-cig vaping system and exposure paradigm were described previously (Orzabal et al., 2019). The vaping chamber system, custom-engineered for thee-cig vaping protocol, comprised a Sense Herakles Sub-Ohm Tank (Sense Technology, San Francisco, CA). Precision-controlled aerosol release technology with a programmable atomizer and airflow control was used to establish the e-cig vaping paradigm. The constant airflow rate of 2.5 L/min through the vaping chambers was controlled with approximately 42 mL of e-cig vapor puff, for 1 s in 20 s intervals. The animals underwent vaping exposure 3 h/day, 5 days/week during pregnancy, from GD 5 to GD 20. The vaping exposure paradigm utilized in this study has been shown to effectively modulate nicotine yield during vaping treatments (Talih et al., 2015).

The e-cig vaping liquid (juice) was compounded with a composition of 80:20 propylene glycol (Fisher, Hampton, NH) to glycerol (Acros Organics, Fair Lawn, NJ), similar to e-cig liquids available on the market. In the present study, we selected a ratio similar to that utilized by human e-cig users to get a “throat hit” sensation, and that has a higher amount of nicotine in the vapor as documented (Li et al., 2016; Ooi et al., 2019; Orzabal et al., 2019, 2022). For the Nicotine group, animals were first acclimatized to lower doses of 5% (50 mg/mL) nicotine added in the same e-cig liquid (juice) during GD 5–8, then treated with higher doses of 10% (100 mg/mL) nicotine during GD 9–20. The 10% nicotine concentration was based on the average nicotine concentration found in e-cigs on the vaping market (Matta et al., 2007; Goniewicz et al., 2013). As described previously, to obtain physiologically relevant levels of serum nicotine and account for differences in human and rodent nicotine metabolism, we previously evaluated the serum nicotine concentrations of dams over a 24 h period (Matta et al., 2007; Orzabal et al., 2022) and reported that the e-cig vaping paradigm utilized in this study produces serum nicotine levels (median peak serum nicotine =27.7 ng/mL), comparable to moderate/high level human smokers (Russell et al., 1980; Benowitz et al., 1983; Benowitz and Jacob, 1984; Lawson et al., 1998; Moyer et al., 2002; Wagener et al., 2017; Wagner et al., 2017; Orzabal et al., 2019). The Nicotine group was utilized to test the effects of vaping e-cig liquid with nicotine. The Juice group was tested for the effects of vaping nicotine-free e-cig liquid (juice). The Control group animals were set in identical conditions to animals in the Juice and Nicotine groups, but with only room air passing through the vaping chambers. We previously reported the chemical composition of the aerosols produced by our vaping system via mass spectrometry that showed several hazardous aldehyde species and other chemicals in addition to propylene glycol and glycerol (Orzabal et al., 2019, 2022). As reported previously (Orzabal et al., 2022), these aerosols resemble the chemical profile of aerosols derived from human e-cig vaping devices (Goniewicz et al., 2013, 2014; Hahn et al., 2014; Talih et al., 2015).

### Fetal brain and hippocampal isolation

On GD 21, a day before parturition, animals—were deeply anesthetized with isoflurane in the anesthesia chamber and sacrificed by decapitation and fetuses were carefully separated. Two male fetuses from each dam were randomly selected from a similar position in the uterine horn. One pup per dam was used for immunoblotting while the other pup was used for immunohistochemistry. During normal development, male and female hippocampal development differ significantly and brain development in humans and animal is directly regulated by sex hormones (Yagi and Galea, 2019; Mu et al., 2020). To accurately identify hippocampal-specific alterations induced by prenatal e-cig aerosol exposure without the confounding factor of sex, only male offspring were assessed in these experiments. Fetal whole brains from the male fetuses were extracted under a dissection microscope via craniotomy and serially washed in cold phosphate buffered saline (PBS). One of the fetal brains was dissected, meninges were removed, and hippocampi were micro-dissected in ice-cold HEPES buffer. Individual samples were then flash frozen and stored at −80°C until immunoblot analyses. For immunohistochemistry, the other whole fetal brain was embedded in optimal cutting temperature compound (OCT) (Sakura Finetek, Torrance, CA) and cryopreserved for immunofluorescence.

### Immunoblotting

Hippocampal tissues were lysed in ice-cold RIPA buffer, pH 7.5 (Cell Signaling Technologies, Danvers, MA) with a protease inhibitor cocktail (cOmplete Mini; Roche Applied Biosciences, Germany). The lysates were centrifuged at 10,000 xg for 10 min at 4°C and supernatants were collected. Total protein concentrations were determined using the Bradford method and a BCA assay kit (Thermo Scientific, Rockford, IL). The protein samples (20 μg) were separated on 4 ~ 20% Tris–HEPES polyacrylamide gels (Bio-Rad, Hercules, CA) by electrophoresis. The separated proteins were transferred onto PVDF membranes (Immobilon-P; Millipore, Bellerica, MA). The membranes were blocked with 5% BSA in 0.01 M PBS (pH 7.4) and 0.05% Tween-20 (TPBS), for 1 h at room temperature. Subsequently, the membranes were probed with one of the following primary antibodies (Cell signaling, Danvers, MA), overnight at 4°C: Phospho-mTOR (Ser2448) (1:1,000; #2971); mTOR (1:1,000; #4517); Phospho-4E-BP1 (Thr37/46) (1:1,000; #2855); 4E-BP1 (1:1,000; #9644); Phospho-p70S6K (Thr389) (1:1,000; #9234); P70S6K (1:1,000; #9202); DEPTOR (1:1,000; #11816); RAPTOR (1:1,000; #2280); RICTOR (1:1,000; #2114); Phospho-PKCα (Thr638/641) (1:1,000; #9375); PKCα (1:1,000; #2056); and β-actin (1:5,000; #4967). The membranes were then incubated with a secondary antibody conjugated to horseradish peroxidase for 1 h at room temperature. The immune complexes were detected by chemiluminescent substrate (Thermo Scientific, Rockford, IL). Densitometry analysis was performed with AzureSpot software (Azure Biosystems, Dublin, CA).

### Immunofluorescence

Fetal brains were sectioned at 8 μm in a coronal plane using a Leica CM1860 cryostat (Leica Biosystems, Buffalo Grove, IL). Sections were fixed with ice-cold methanol (30 min, −20°C), rinsed in PBS, incubated in 10% normal serum (60 min), followed by incubation with Phospho-mTOR (Ser2448) (1:250; #2971) primary antibody overnight at 4°C in a humidified chamber. The sections were further incubated with goat anti-rabbit IgG secondary antibody (Alexa Fluor 488, #A11008; Invitrogen, Carlsbad, CA), for 60 min at room temperature. Nuclei were stained with DAPI (ProLong Gold antifade, #P36931; Invitrogen, Carlsbad, CA). Digital images were captured using an Olympus BX63 stereomicroscope equipped with U-HGLGPS fluorescent light source, ORCA-Flash 4.0 LT digital camera (Hamamatsu, Japan), and Olympus cellSens Dimension 1.16 software (Olympus, Japan).

### Statistics

Immunoblotting data for the expression levels of different proteins were analyzed utilizing one-way ANOVA with a *post hoc* analysis of uncorrected Fisher’s LSD. Normality was assessed using Shapiro–Wilk normality test, and dependent measurements passed normality prior to performing ANOVA. All data are presented as mean ± SEM. Significance was established *a priori* at *p* < 0.05.

## Results

Gestational e-cig vaping in our model results in reduced fetal weight and fetal crown-rump length, in the absence of any changes to maternal weight as reported previously (Orzabal et al., 2019, 2021, 2022). These growth deficits were specific to the nicotine-containing group and were not observed following vaping juice in the absence of nicotine (Orzabal and Ramadoss, 2019).

**Figure 1:**
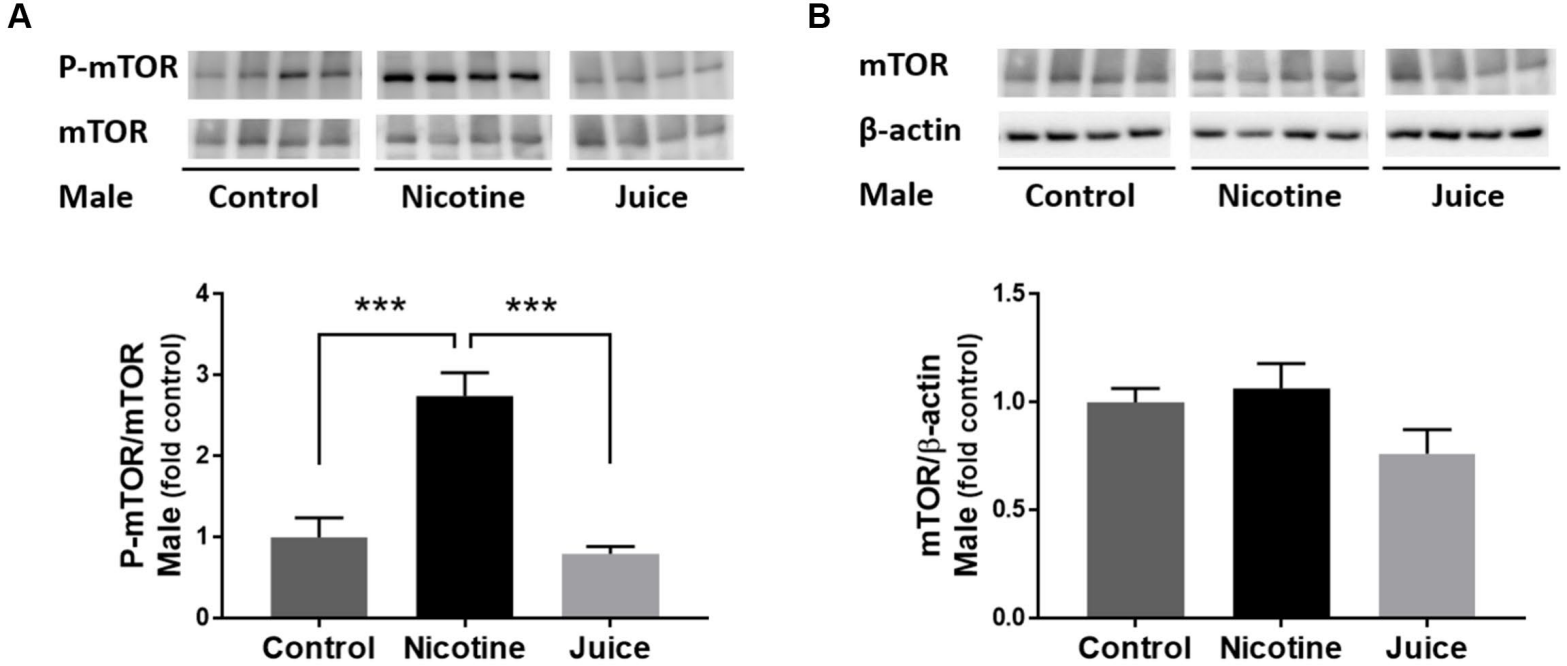
Effect of gestational e-cig vaping exposure on fetal hippocampal mTOR. **(A)** Immunoblot analysis showed that gestational e-cig vaping exposure significantly (***) increased (↑ 274%; Control vs. Nicotine; *p* = 0.0002 and ↑347%; Nicotine vs. Juice; *p* = 0.0001) P-mTOR levels in the fetal hippocampi. **(B)** Total mTOR expression was not different between groups. Data are shown as mean ± SEM. Significance was established *a priori* at *p* < 0.05.

We analyzed the effect of prenatal chronic exposure to e-Cig nicotine on mTOR protein expression in the fetal hippocampus. Immunoblot analysis showed that the phosphorylation level of mTOR (P-mTOR) in fetal hippocampi was significantly higher in the Nicotine group, compared with those in the Control group [↑ 274%; Control vs. Nicotine; *t*(11) = 5.392, *p* = 0.0002] and the Juice group [↑ 347%; Nicotine vs. Juice; *t*(11) = 5.674, *p* = 0.0001; Figure 1A]. In comparison to the Control group, the treatment in the Juice group did not affect the level of P-mTOR (*p* = 0.57; Figure 1A). There was no difference in total mTOR level among the Control, Nicotine, and Juice groups (Figure 1B).

**Figure 2:**
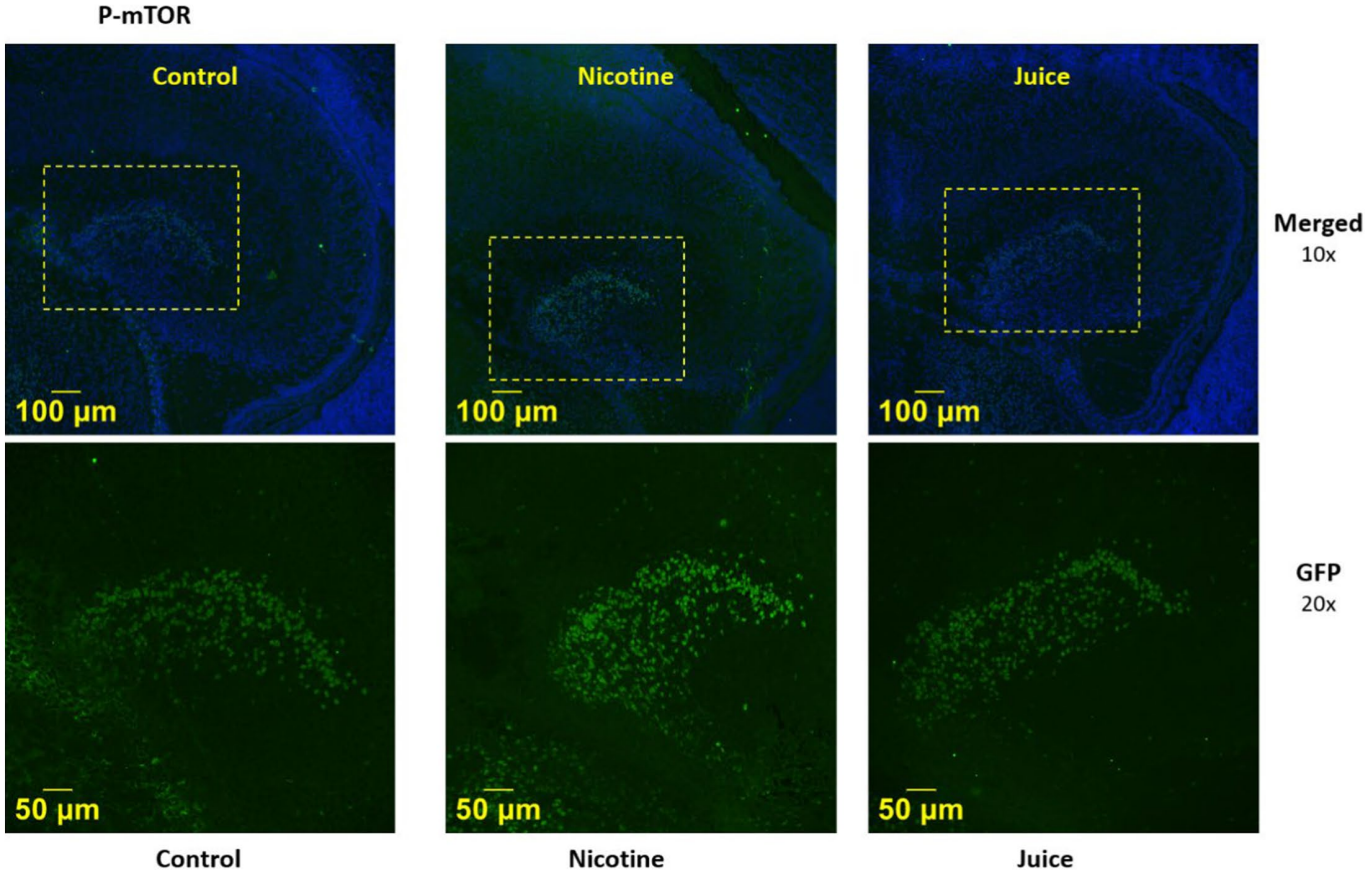
Effect of gestational e-cig vaping on fetal hippocampal Dentate Gyrus mTOR. Representative immunofluorescence staining shows P-mTOR was expressed in the fetal dentate gyrus (DG) region.

We then imaged the presence of activated mTOR (P-mTOR) using immunofluorescence microscopy. Immunofluorescence imaging (Figure 2) demonstrated that P-mTOR was detected exclusively in the granule cells of the dentate gyrus of the fetal hippocampus in Nicotine group, compared to both Control and Juice groups. Collectively, these findings suggest that mTOR phosphorylation in the fetal hippocampus increases following gestational chronic nicotine exposure.

**Figure 3:**
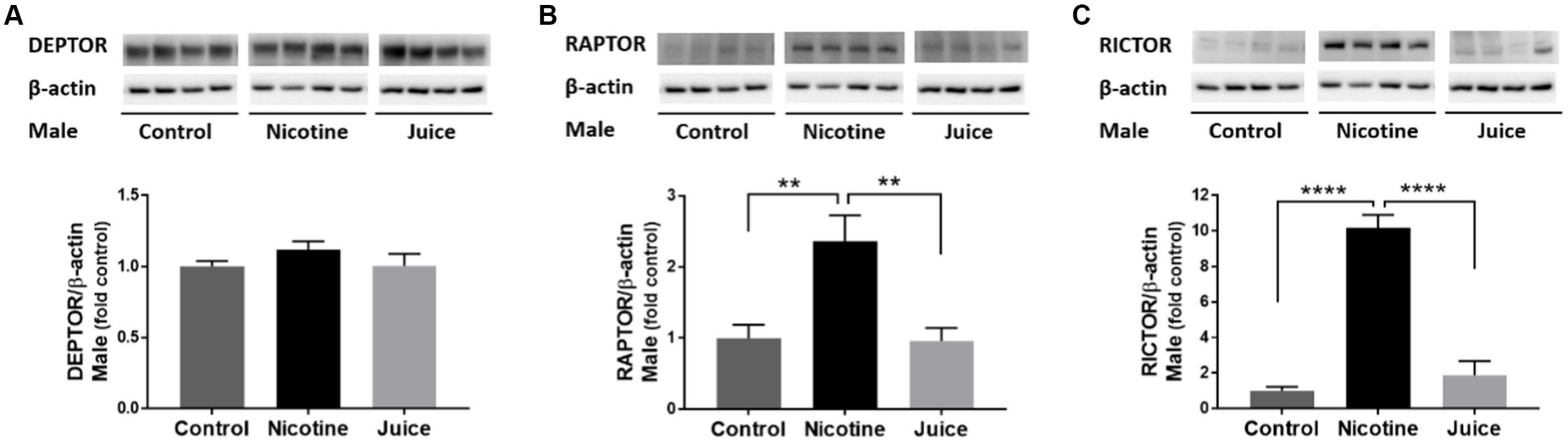
Effect of gestational e-cig vaping exposure on the proteins that complex with hippocampal mTOR. Immunoblot analysis showed that gestational E-Cig vaping exposure **(A)** did not alter DEPTOR expression, but **(B,C)** expression levels of RAPTOR (**) and RICTOR (****) were significantly higher in the Nicotine group compared to those of Control and Juice groups (RAPTOR, ↑ 136%; Control vs. Nicotine, *p* = 0.0038; RICTOR, ↑ 1,017%; Control vs. Nicotine, *p* < 0.0001). DEPTOR, RAPTOR, and RICTOR proteins were normalized to β-actin and were expressed relative to Control. Data are shown as mean ± SEM. Significance was established *a priori* at *p* < 0.05.

To investigate the effect of gestational chronic nicotine exposure on mTOR complexes, we analyzed hippocampal levels of DEPTOR, RAPTOR, and RICTOR (Figure 3). The DEPTOR expression level was not affected by prenatal chronic exposure to e-cig nicotine. Quantitative analysis showed that the expression levels of RAPTOR and RICTOR were significantly higher in the Nicotine group compared to those of Control and Juice groups [RAPTOR, ↑ 136%; Control vs. Nicotine; *t*(11) = 3.657, *p* = 0.0038 and RICTOR, ↑ 1,017%; Control vs. Nicotine; *t*(11) = 10.87, *p* < 0.0001]. Distinctly, the RAPTOR expression level increased over 10-fold in the Nicotine group compared to Control. There were no significant differences between Control and Juice groups in RAPTOR or RICTOR levels.

**Figure 4:**
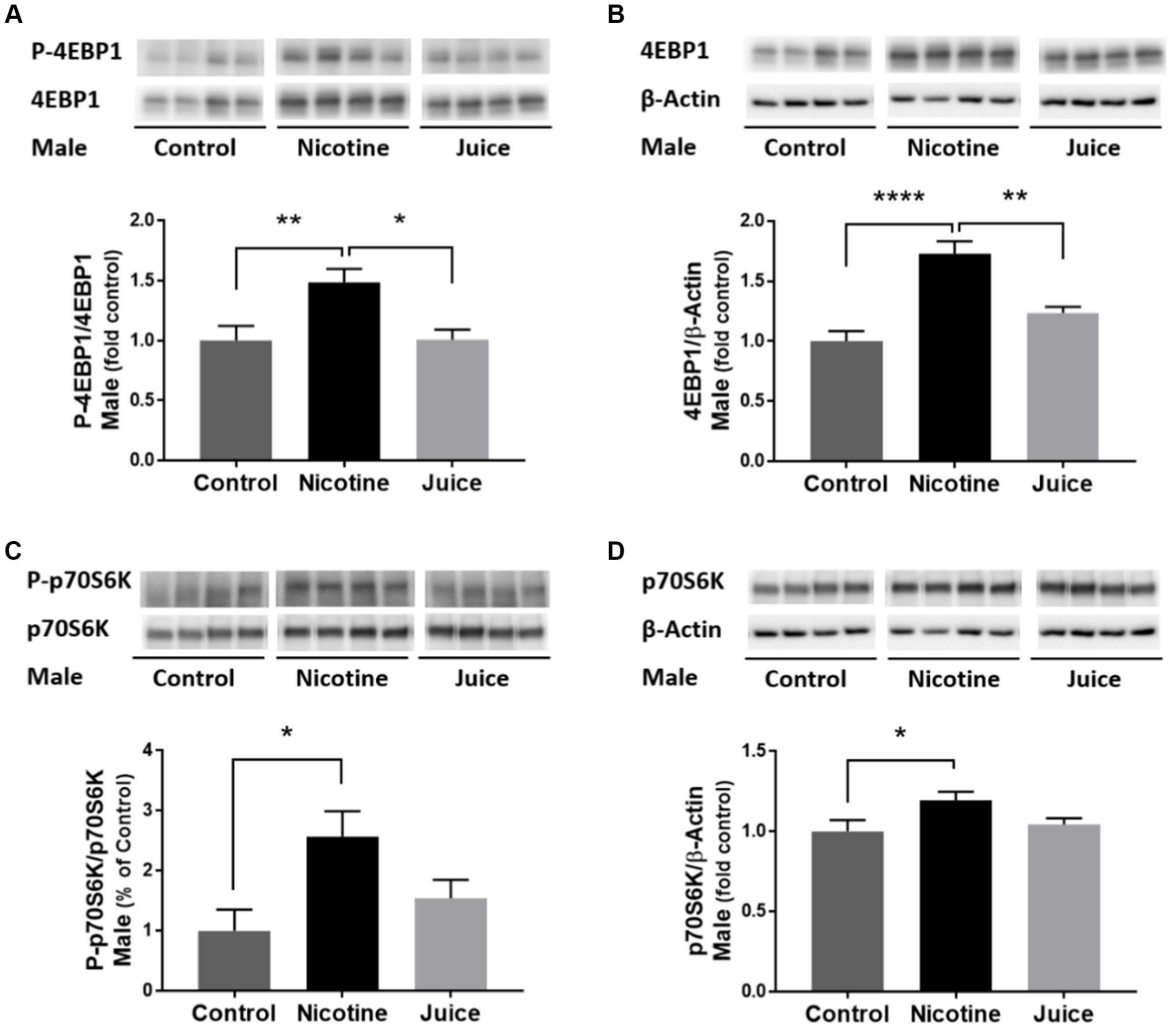
Effect of gestational e-cig vaping exposure on mTORC1 signaling in the fetal hippocampus. Gestational e-cig vaping **(A)** significantly increased the phosphorylation level of 4EBP1 (P-4EBP1) (Control vs. Nicotine, *p* = 0.0084) in the fetal hippocampi from the Nicotine group compared to the Control group (**) and the Juice group (*). **(B)** Hippocampal total 4EBP1 expression also significantly increased (Control vs. Nicotine, *p* < 0.0001) in the Nicotine group compared to that of the Control group (****) and the Juice group (**). **(C)** Hippocampal phosphorylation of p70S6K (P-p70S6K) increased (Control vs. Nicotine, *p* = 0.0109) in the Nicotine group compared to the Control group (*). **(D)** Total p70S6K level was also higher (Control vs. Nicotine; *p* = 0.0340) in the Nicotine group compared to the Control group (*). There were no significant differences between the Control and Juice groups in P-4EBP1, 4EBP1, P-p70S6K, or p70S6K levels. Data are shown as mean ± SEM and as fold of control. Significance was established *a priori* at *p* < 0.05.

To determine if gestational chronic nicotine exposure dysregulates mTORC1 signaling, we analyzed the abundance levels of downstream molecules within mTORC1 signaling pathway. The phosphorylation level of 4EBP1 (P-4EBP1) was significantly higher [↑ 49%; Control vs. Nicotine; *t*(11) = 3.204, *p* = 0.0084] in the fetal hippocampi from the Nicotine group compared to the Control group (Figure 4A). Hippocampal total 4EBP1 expression also significantly increased [↑ 73%; Control vs. Nicotine; *t*(11) = 6.154, *p* < 0.0001] in the Nicotine group compared to that of the Control group (Figure 4B). Hippocampal phosphorylation of p70S6K (P-p70S6K) increased [↑ 256%; Control vs. Nicotine; *t*(11) = 3.059, *p* = 0.0109] in the Nicotine group compared to the Control group (Figure 4C). Total p70S6K Total p70S6K level was also higher [↑ 19%; Control vs. Nicotine; *t*(11) = 2.419, *p* = 0.0340] in the Nicotine group compared to the Control group (Figure 4D). There were no significant differences between the Control and Juice groups in P-4EBP1, 4EBP1, P-p70S6K, or p70S6K levels.

**Figure 5:**
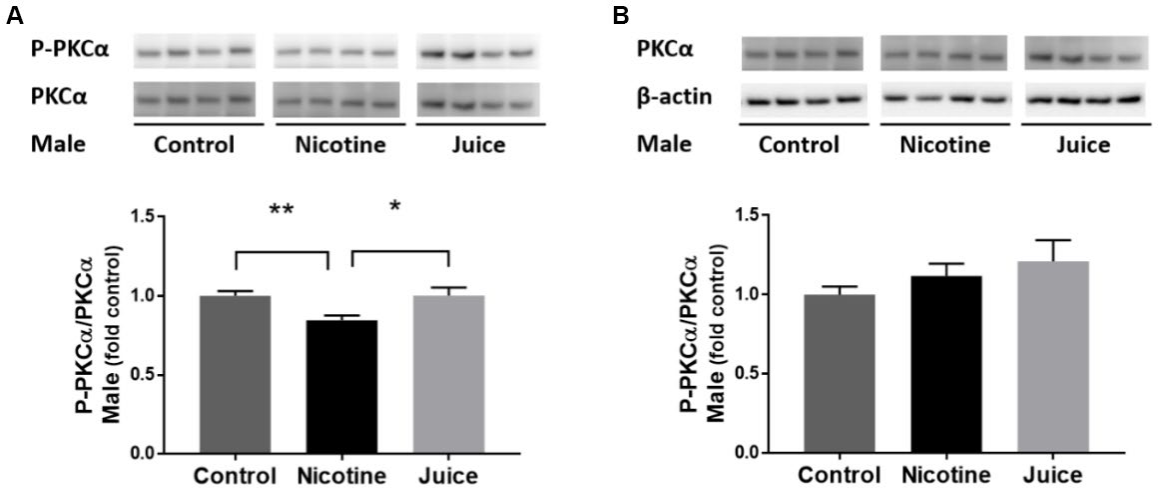
Effect of gestational e-cig vaping exposure on mTORC2 signaling in the fetal hippocampus. Gestational e-cig vaping **(A)** decreased the phosphorylation level of PKCα (P-PKCα) (Control vs. Nicotine; *p* = 0.0098) in the fetal hippocampi from the Nicotine group compared to that of the Control group (**) and the Juice group (*). In comparison to the Control group, the treatment in the Juice group did not affect the level of P-PKCα (*p* = 0.79). **(B)** No significant difference was observed in total PKCα levels among groups. Data are shown as mean ± SEM and as fold of control. Significance was established *a priori* at *p* < 0.05.

To determine if gestational chronic nicotine exposure dysregulates the downstream of mTORC2 signaling pathway, we analyzed the protein kinase C alpha (PKCα). The phosphorylation level of PKCα (P-PKCα) was significantly lower [↓ 15%; Control vs. Nicotine; *t*(11) = 3.115, *p* = 0.0098] in the fetal hippocampi from the Nicotine group compared to that of the Control group (Figure 5A). In comparison to the Control group, the treatment in the Juice group did not affect the level of P-PKCα (*p* = 0.79; Figure 5A). No significant difference was observed in total PKCα levels among groups (Figure 5B).

## Discussion

**Figure 6:**
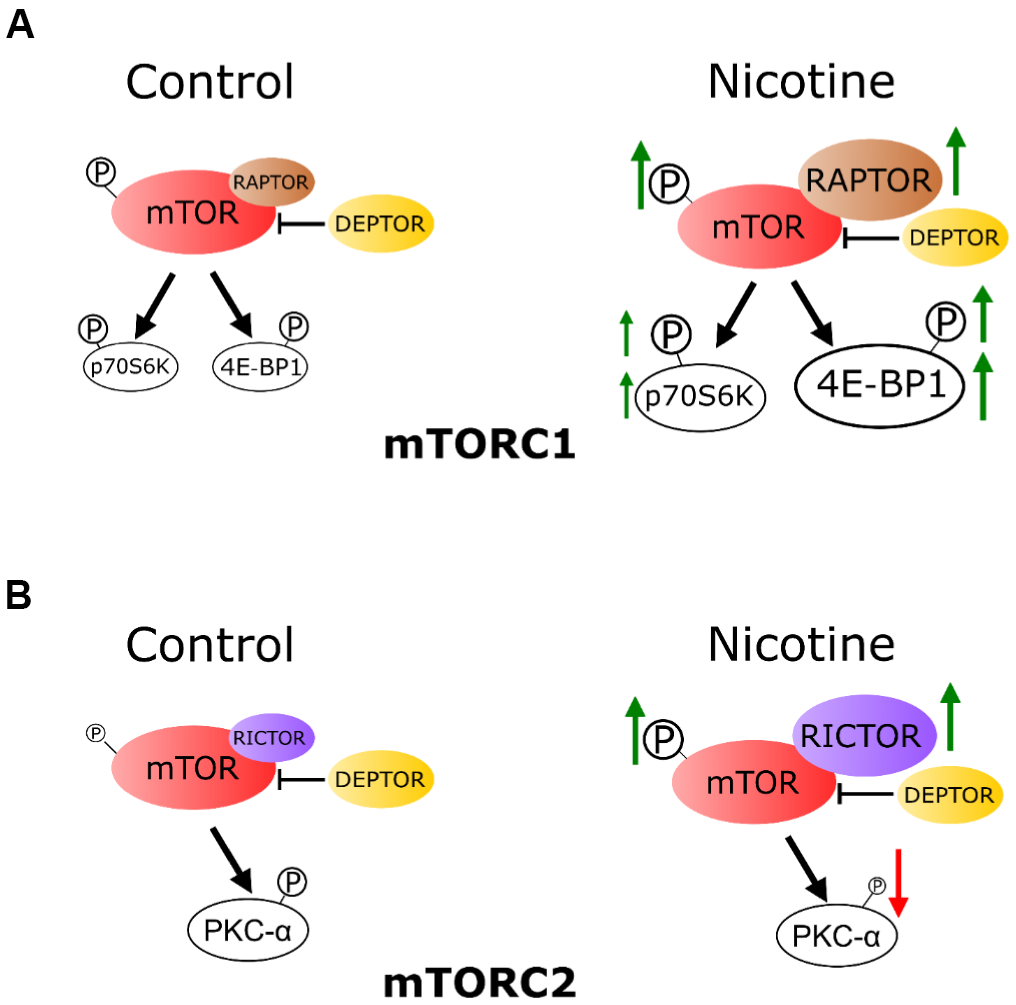
Schematic representation: chronic e-cig vaping exposure during pregnancy alters mTOR system in rat fetal hippocampus. **(A)** The phosphorylation level of mTOR (P-mTOR) in the fetal hippocampus was increased in the e-cig vaping group compared with controls. These effects were mainly due to nicotine component of the e-cig vaping juice. Vaping dysregulated fetal hippocampal mTORC1 signaling, as evidenced by an increase in 4E-BP1 expression. Phosphorylation levels of 4E-BP1 and p70 S6K were also increased following e-cig exposure. RAPTOR and RICTOR expression levels in the fetal hippocampus were increased, however DEPTOR was not altered by e-cig vaping. **(B)** E-cig vaping also altered mTORC2 system as evidenced by increased RICTOR and decreased phosphorylated PKC-α levels in the fetal hippocampus.

To our knowledge, this is the first investigation of e-cig vaping effects on fetal hippocampal mTOR signaling following a gestational chronic vaping exposure paradigm. Four findings can be gathered from this study: gestational e-cig vaping (1) dysregulates the fetal hippocampal mTOR system, (2) has an impact on both mTORC1 and mTORC2 signaling, which plays critical roles in hippocampal development, (3) alters the proteins that complex with fetal hippocampal mTOR and the respective downstream pathways, and (4) the changes were related to nicotine component in the e-cig vape juice (Figure 6). Together, these findings suggest that gestational e-cig vaping exposure alters mTOR signaling framework, activity indices, and associated downstream signaling in the fetal hippocampus, and these vaping-induced alterations may contribute to altered hippocampal developmental adaptations.

### Gestational e-cig vaping alters fetal hippocampal mTOR

Our data showed that e-cig vaping during gestation altered the mTOR system. Although little is known about the effects of e-cig exposure on the mTOR system in the developing brain, there are reports documenting e-cig effects on other organ systems (Orzabal et al., 2019, 2022; Orzabal and Ramadoss, 2019). The mTOR signaling system is intricately associated with fetal and neonatal brain development and is essential for growth, survival, and differentiation of neuronal stem and progenitor cells (Saxton and Sabatini, 2017). Normal development of brain size is also controlled by mTOR system and mice lacking mTOR exhibit smaller brain size with decreased neurons and neuronal progenitor cells (Kim, 2015). Although studies on brain mTOR in the context of e-cigarette vaping are limited, others have reported effects of e-cig vaping on mTOR in other organs systems. For instance, one study using pluripotent stem cell-derived endothelial cells (iPSC-ECs) from healthy individuals and patients with pulmonary arterial hypertension (PAH) showed differential susceptibility of PAH group to e-cig bubbling and demonstrated pharmacologic induction of autophagy via direct inhibition of mTORC1 and indirect activation of mTORC2 with rapamycin reversing the e-cig-induced downstream effects on endothelial dysfunction and AKT3 signaling (Liu et al., 2023). We had previously reported that e-cig vaping during gestation results in an increase in the concentration of the branch chain amino acids (leucine, isoleucine, valine) in the rat fetal lungs (Orzabal et al., 2021), and others have shown that the increases in branch chain amino acids results in activation of mTORC1, which in turn modulates the production of reactive oxygen species and leads to an inflammatory phenotype (Zhenyukh et al., 2018). Although the current study did not assess relationship between mTOR system and brain structural and functional alterations following e-cig vaping during pregnancy, others have utilized female CD1 pregnant mice and demonstrated that prenatal e-cig vaping decreases brain glucose utilization and worsens the outcome in offspring hypoxic–ischemic brain injury, but did not specifically assess the mTOR pathway (Sifat et al., 2020). Outside the e-cig research area, mutations of mTOR have been associated with a spectrum of brain overgrowth phenotypes (Mirzaa et al., 2016).

### E-cig vaping has an impact on both mTORC1 and mTORC2 signaling pathways

To determine if gestational chronic nicotine exposure dysregulates mTORC1 signaling, we analyzed the abundance levels of downstream molecules within mTORC1 signaling pathway. The phosphorylation level of 4EBP1 (P-4EBP1) and total 4EBP1 expression were both significantly higher in the fetal hippocampi from the Nicotine group. Hippocampal phosphorylation of p70S6K (P-p70S6K) and Total p70S6K increased in the Nicotine group compared to the Control group. To determine if gestational chronic nicotine exposure dysregulates the downstream of mTORC2 signaling pathway, we analyzed the protein kinase C alpha (PKCα). The phosphorylation level of PKCα (P-PKCα) was significantly lower in the fetal hippocampi from the Nicotine group. mTOR makes up the catalytic subunit of two complexes, mTORC1 and mTOR2, each of which have distinctive substrates and functions, and unique accessory proteins and sensitivity to rapamycin (Yang et al., 2013; Zarogoulidis et al., 2014; Liu and Sabatini, 2020). mTORC1 plays a major role in convergence of multiple cellular signals to sense the availability of nutrients, growth factors, and energy to stimulate cellular growth and catabolic activity during stress (Liu and Sabatini, 2020). In contrast, little is known about mTORC2 although it is possible that mTORC2 may have some regulating effect on mTORC1 system (Liu and Sabatini, 2020; Szwed et al., 2021). mTORC1 directly promotes protein synthesis by ultimately phosphorylating the Eukaryotic Translation Initiation Factor 4E-binding Proteins (4E-BPs), as well as Ribosomal Protein S6 Kinase 1 (S6K1; Carabulea et al., 2023). Others have shown that nicotine significantly reduced hyperalgesia in mice that received acute/repeated rapamycin injections, and nicotine reversed the outcomes of rapamycin on the phosphorylation of 4E-BP1 and S6K (Li S. et al., 2019; Li Y. et al., 2019). Outside the e-cig field, secondhand smoke during pregnancy showed decreased activation of the mTOR family of proteins in mice placenta, and these may be explained by differences between smoking and vaping and also the tissue being studied, i.e., placenta vs. the brain in the current study (Mejia et al., 2017). In another study, the human brain microvascular endothelial cell line was cultured, and nicotine treatment significantly activated the PI3K/Akt/mTOR pathway (Wu et al., 2020). Overall, this is the first report to demonstrate that the nutrient-sensing mTORC1 and the mTORC2 pathways are both altered by e-cig vaping during development.

### E-cig alters the proteins that complex with mTOR

To investigate the effect of gestational chronic nicotine exposure on mTOR complexes, we analyzed hippocampal levels of DEPTOR, RAPTOR, and RICTOR. The DEPTOR expression level was not affected by prenatal chronic exposure to e-Cig nicotine. The expression levels of RAPTOR and RICTOR were significantly higher in the Nicotine group compared to those of Control. Distinctly, the RAPTOR expression level increased over 10-fold in the Nicotine group compared to Control. RAPTOR is essential for intracellular localization of mTORC1 and has a role in recruiting substrates by binding to respective mTOR signaling motifs and the core of mTORC2 is instead defined by the scaffold protein, RICTOR (Carabulea et al., 2023). There is an overall lack of information available about the effects of e-cig on RICTOR, RAPTOR, and DEPTOR in any organ system, including the brain, especially in development. This study fills a major gap in the literature and provides critical information of brain hippocampal effects of e-cig vaping.

### The role of nicotine component of e-cig vape juice in developmental adaptations

e-liquids are not standardized by manufacturers (Smith et al., 2020). VG (40 CFR 180.950) and PG (21 CFR 184.1666) are both generally regarded as safe for oral ingestion as liquids by the FDA and EPA (GRAS), whereas aerosolized VG/PG has not been classified as safe(Qu et al., 2018; Berkelhamer et al., 2019; Frazier et al., 2021). Currently, the proportion of VG:PG is chosen based on personal preferences for the sensory profiles VG and PG contribute. Flavor chemicals are a major component of e-cig refill fluids (Behar et al., 2018; Hua et al., 2019; Krusemann et al., 2020), but the current study did not use flavorings. The data showed that at least as far as mTOR in fetal hippocampus is concerned, neither VG nor PG played a role, and the alterations were nicotine dependent. Nicotine in vapor form has been reported to have a range of effects on reproductive function, fetal and child development such as low birth weight, increased blood pressure, and increase in inflammatory phenotype in the newborn (Suzuki et al., 1980; Aubert et al., 1987; Mohsenzadeh et al., 2014). In contrast to our data, one study that examined effects of dihydroxyacetone, a combustion production of propylene glycol and glycerol in e-cig vapor on human hepatocellular carcinoma cell line, showed slight increases in p-mTOR, while mTOR, RICTOR and RAPTOR displayed fluctuating levels (Hernandez et al., 2022); these differences could be due to the levels of dihydroxyacetone in an *in vitro* setting.

### Perspectives and significance

The effect of e-cig vaping on the hippocampal mTOR signaling in the context of pregnancy and development is largely unknown. We utilized an established gestational e-cig vaping paradigm to delineate how vaping impacts the developmental hippocampal mTOR signaling system. Based on our data, we conclude that gestational e-cig vaping exposure alters mTORC1 and mTORC2 signaling, their activity indices, and related downstream pathways in the developing hippocampus. Although we report deficits in fetal body weight and length, we did not examine neuroanatomic and structural deficit; future studies are warranted to assess the structural, functional, and behavioral deficits that arise out of developmental e-cig vaping exposure and if and how they relate to mTOR signaling in hippocampus. These studies may in turn facilitate development of clear targeted pharmacological and nutraceutical therapeutic strategies for any developmental deficits related to hippocampal functions of learning and memory during development.

## Data availability statement

The raw data supporting the conclusions of this article will be made available by the authors, without undue reservation.

## Ethics statement

All experimental procedures were in accordance with National Institutes of Health guidelines (NIH Publication No. 85–23, revised 1996), with approval by the Animal Care and Use Committee at Texas A&M University.

## Author contributions

JL and JR: conceptualization, methodology, writing—original draft, writing—review and editing, visualization. JL, MO, VN, and JR: investigation. JR: funding acquisition, project administration, and supervision. All authors contributed to the article and approved the submitted version.

## Funding

This study was supported by the National Institutes of Health (Nos. HL151497, AA23520, and, AA23035) to JR.

## Conflict of interest

The authors declare that the research was conducted in the absence of any commercial or financial relationships that could be construed as a potential conflict of interest.

## Publisher’s note

All claims expressed in this article are solely those of the authors and do not necessarily represent those of their affiliated organizations, or those of the publisher, the editors and the reviewers. Any product that may be evaluated in this article, or claim that may be made by its manufacturer, is not guaranteed or endorsed by the publisher.

## Abbreviations

e-cig: Electronic cigarette
GD: gestational day
Juice: e-cig aerosol vaping without nicotine
Nicotine: e-cig aerosol vaping with nicotine
VG: vegetable glycerin
PG: propylene glycol
